# Adding glycemic and physical activity metrics to a multimodal algorithm-enabled decision-support tool for type 1 diabetes care: Keys to implementation and opportunities

**DOI:** 10.3389/fendo.2022.1096325

**Published:** 2023-01-12

**Authors:** Dessi P. Zaharieva, Ransalu Senanayake, Conner Brown, Brendan Watkins, Glenn Loving, Priya Prahalad, Johannes O. Ferstad, Carlos Guestrin, Emily B. Fox, David M. Maahs, David Scheinker

**Affiliations:** ^1^ Division of Endocrinology, Department of Pediatrics, Stanford University, School of Medicine, Stanford, CA, United States; ^2^ Department of Computer Science, Stanford University, Stanford, CA, United States; ^3^ Stanford Children’s Health, Lucile Packard Children’s Hospital, Stanford, CA, United States; ^4^ Stanford Diabetes Research Center, Stanford University, Stanford, CA, United States; ^5^ Department of Management Science and Engineering, Stanford University School of Engineering, Stanford, CA, United States; ^6^ Chan Zuckerberg Biohub, San Francisco, CA, United States; ^7^ Department of Statistics, Stanford University, Stanford, CA, United States; ^8^ Department of Health Research and Policy, Stanford University School of Medicine, Stanford, CA, United States; ^9^ Clinical Excellence Research Center, Stanford University School of Medicine, Stanford, CA, United States

**Keywords:** type 1 diabetes, algorithm-support, exercise, continuous glucose monitoring (CGM), remote monitoring

## Abstract

Algorithm-enabled patient prioritization and remote patient monitoring (RPM) have been used to improve clinical workflows at Stanford and have been associated with improved glucose time-in-range in newly diagnosed youth with type 1 diabetes (T1D). This novel algorithm-enabled care model currently integrates continuous glucose monitoring (CGM) data to prioritize patients for weekly reviews by the clinical diabetes team. The use of additional data may help clinical teams make more informed decisions around T1D management. Regular exercise and physical activity are essential to increasing cardiovascular fitness, increasing insulin sensitivity, and improving overall well-being of youth and adults with T1D. However, exercise can lead to fluctuations in glycemia during and after the activity. Future iterations of the care model will integrate physical activity metrics (e.g., heart rate and step count) and physical activity flags to help identify patients whose needs are not fully captured by CGM data. Our aim is to help healthcare professionals improve patient care with a better integration of CGM and physical activity data. We hypothesize that incorporating exercise data into the current CGM-based care model will produce specific, clinically relevant information such as identifying whether patients are meeting exercise guidelines. This work provides an overview of the essential steps of integrating exercise data into an RPM program and the most promising opportunities for the use of these data.

## Introduction

The Diabetes Control and Complications Trial (DCCT) and the Epidemiology of Diabetes Interventions and Complications (EDIC) follow-up study have established the importance of intensive diabetes management in decreasing the development and progression of microvascular and neurologic complications (1, 2). In addition to intensive insulin therapy, increased levels of physical activity are shown to improve cardiovascular fitness and strength, increase insulin sensitivity, improve blood lipid profiles, and overall well-being in individuals with type 1 diabetes (T1D) (3). Maintaining glycemia in a targeted glucose range during and after exercise can be challenging for many individuals with T1D. Numerous factors need to be taken into consideration prior to engaging in physical activity such as the timing, duration, and intensity of exercise, insulin adjustments, nutrition, and the amount of active insulin present at the onset of exercise. If these factors are ignored and not managed accordingly, there is an increased risk of developing hypoglycemia or hyperglycemia during and after exercise. In fact, fear of hypoglycemia and the lack of knowledge on how to exercise safely are some of the leading barriers to exercise in adults and youth with T1D (4–6).

Exercise consensus guidelines recommend that children and adolescents should aim to achieve at least 60 minutes of daily physical activity (7–9). However, research has demonstrated that around 2/3 of youth with T1D were not participating in even 30 minutes of daily physical activity (10). In addition, young children with T1D were also found to be less active and engage in fewer minutes of moderate-intensity physical activity compared to their counterparts without T1D (11). This may be due to exercise increasing the likelihood of glucose excursions, particularly hypoglycemia, and the practical difficulties around glucose monitoring. In overcoming some of these patient-facing challenges with exercise and diabetes management, healthcare professionals need to actively promote physical activity and offer guidance around safe exercise.

Healthcare professionals, however, have also identified numerous barriers to physical activity promotion including competing demands at work, lack of confidence in physical activity knowledge, and accessibility to exercise guidelines and resources (12, 13).

Physical activity metrics (e.g., wearable activity tracker data) are not commonly integrated into standard of care clinical diabetes visits to help with physical activity promotion. Our aim is to integrate wearable physical activity tracker data (e.g., heart rate, step count, etc.) into an existing algorithm-enabled decision-support tool to help promote physical activity in newly diagnosed youth with T1D and improve clinical outcomes.

### Current TIDE care model and the 4T study

An open-source algorithm-enabled care model providing patient prioritization, called Timely Interventions for Diabetes Excellence (TIDE), was developed at Stanford to support clinicians and Certified Diabetes Care and Education Specialists (CDCES) at Stanford Children’s Pediatric diabetes clinic (14–16). The current TIDE care model facilitates population-level algorithm-enabled remote patient monitoring (RPM) based on continuous glucose monitoring (CGM) data (15). TIDE is being used as a part of the larger R18-funded pragmatic research study called the Teamwork, Targets, Technology, and Tight Control 4T Study (17–21). Based on feedback from CDCES’, other team members, and analysis of historical patient data, TIDE is constantly being updated with improvements to the visual interface, the algorithmic ranking, and to better fit the clinic workflows (14).

### CGM metrics and accessing glucose data

In the current 4T Study, all newly diagnosed youth with T1D are started on a CGM system (Dexcom G6, DexCom Inc., San Diego, CA) and remote CGM review within the first month of diagnosis. The CGM system measures up to 288 interstitial glucose readings per day and transmits data to the Dexcom app using Bluetooth. This glucose data is automatically transmitted to Dexcom’s remote database when the mobile phone or smart device is connected to the internet. An internally developed Python-based software tool that makes use of the Dexcom Application Programming Interface (API) is used to download data from Dexcom’s remote database to an internal database of the Lucile Packard Children’s Hospital (LPCH). This downloaded data for all participants is then aggregated and processed to compute the metrics of interest. Based on the consensus guidelines on clinical targets for CGM (22), the following metrics are computed and integrated into the TIDE platform: percent CGM wear time, mean glucose (mg/dL), percent time-in-range (TIR; 70-180 mg/dL), percent change in TIR from the previous review period, percent time in hypoglycemia (<70 mg/dL and <54 mg/dL), percent time in hyperglycemia (>180 mg/dL and >250 mg/dL). TIDE is hosted on LPCH’s servers and the CDCES team can access it from their office. TIDE indicates which participants to prioritize for review and shows daily CGM traces over the previous two weeks.

### Activity metrics and accessing physical activity data

In the current 4T Exercise Study, a subset of participants from the main 4T Study (11+ years of age) are also started on a physical activity tracker (Garmin vívosmart^®^ 4 or Garmin Venu^®^ Sq) around one month following T1D diagnosis and CGM start. Participants in the study are trained to use the watch and instructed to wear it throughout the 12 month study duration, with the goal of capturing at least two weeks of activity data each month (17). The activity trackers capture data including, but not limited to, heart rate, activity type, active kilocalories, step count, stress, metabolic equivalents (METS), etc. The overall aim is for the activity trackers to provide the clinical team with insights into the activity patterns, trends, and behaviors of newly diagnosed youth with T1D.

To collect and access the physical activity data, the study staff first create individual de-identified study accounts in Garmin Connect for all newly enrolled 4T Exercise participants. The physical activity data are transmitted from the activity tracker to the Garmin Connect application using Bluetooth. To get access to the raw participant data files, we use the Garmin Developer API and have an application hosted in the cloud that receives the data. The first step was to create a Garmin Developer account and get approved for API access credentials. This required setting up a test integration and completing a review from the Garmin developers. This is a one-time step in order to use the Garmin Developer API. For our application to receive data for any given Garmin Connect account, we then complete a data access authorization flow with Garmin between the Garmin Connect account and our API credentials. This requires both the API credentials and the Garmin Connect account credentials and this authorization is completed in our application. At this point, we have participants with Garmin watches, which are connected to a Garmin Connect account that we have created for them, and all of the data that gets tracked by the Garmin account has been authorized to be sent to our application. As our application receives Garmin data, we store all of the raw data that we receive. When the raw data are downloaded from our application, we make sure to remove any duplicate or incomplete data entries that Garmin has sent.

There are additional features that our application allows, including: unauthorizing a participant’s Garmin Connect account from our API credentials so we will no longer receive data; backfilling participant data (in the event that we missed an event from Garmin and getting access to two years of historic account data); reporting on the last day that data was received for a participant; and reporting on approximate watch wear time (estimated by analyzing the Garmin heart rate data that has been received).

### Data processing and data flow into TIDE

TIDE is deployed within the secure data infrastructure of the Lucile Packard Children’s Hospital and through HIPAA-compliant web interfaces and encrypted data storage servers. Our team at Stanford has developed a software program to automatically pull raw data from the remote databases of Dexcom and Garmin daily, at midnight. This allows the care team to have access to updated data files in the morning and can plan their day accordingly (i.e., which patients to contact). We then synchronize the two data streams and visualize both CGM and physical activity data in TIDE, locally. We have data coming from two independent sources: Dexcom’s remote database and Garmin’s remote database. We independently download Dexcom data as a comma-separated values (csv) file and Garmin data as a JavaScript Object Notation (JSON) file onto a local computer (i.e., we are currently running this data integration on a testing environment) as described in sections “*CGM Metrics and Accessing Glucose Data*” and “*Activity Metrics and Accessing Physical Activity Data*”, respectively. We developed a Python program to synchronize these two sources of data based on the Standard time. CGM glucose data is available every 5 minutes, however, there is no regular interval for Garmin data as it captures various measurements (e.g., heart rate, type of activity, and intensity of activity) which are aggregated at differing time intervals.

**Figure 1:**
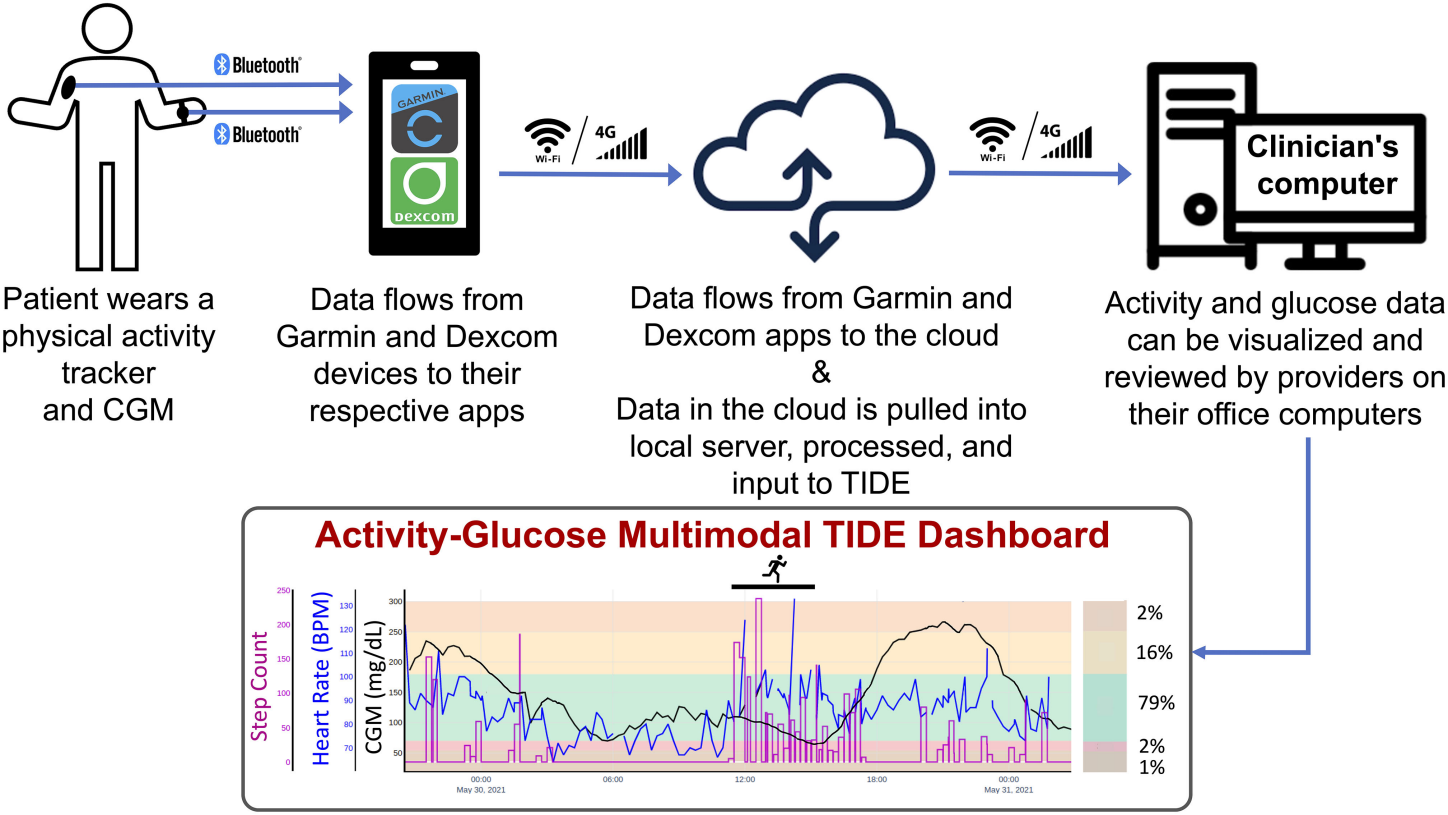
Multimodal decision-support Timely Interventions for Diabetes Excellence (TIDE) care model integrating continuous glucose monitoring (CGM) and physical activity (Garmin Vivosmart 4 or Venu Sq) metrics.

We are currently overlaying CGM, heart rate, and step count values locally, as shown in **Figure 1**. CGM and heart rate values are shown as line plots and a linear interpolation is performed to create smoother plots for visualization. The step count is shown as a step plot. If there are missing values for more than 15 minutes for any particular metric, then we do not include missing values for that particular metric. For instance, if a participant has taken off the activity tracker for a period of time (e.g., 1 hour), then only the CGM values for that 1-hour period will be displayed. Currently, we are focusing on the integration of activity types that contain step counts (e.g., walking, running, etc.), but we intend to expand this further to include all physical activity data types. The intensity of the activity is computed over a period of 15 minutes (called an *Epoch summary* based on Garmin’s Health REST API Specification document) and categorized into three measures: “sedentary”, “active”, or “highly active”. Epoch summaries contain information about wellness data, including steps and distance, and are broken down into short time periods (i.e., 15 minutes). The synchronized, combined Dexcom-Garmin data are converted to a Pandas data frame in Python and provided as the input to TIDE.

### Future directions and use cases with the TIDE care model

To date in the 4T Exercise Study, we have been collecting and monitoring participant’s physical activity data offline (i.e., not in real-time). This physical activity data is also reviewed and used to provide specific exercise education to newly diagnosed youth with T1D in the 4T Exercise Study, led by a Certified Exercise Physiologist. The next steps are to take the physical activity metrics (i.e., heart rate and step count) overlayed with CGM data live in future iterations of the TIDE care model. Our team has chosen to start with heart rate and step count metrics to integrate into TIDE as these are additional signals commonly considered for exercise detection with closed loop technology (23–25). This data will be displayed in TIDE for healthcare providers and CDCES’ to visualize participants’ physical activity data patterns and allow more exercise-specific discussions during standard diabetes care visits (**Figure 1**). These activity workflows and visualizations are being developed with support from a Certified Exercise Physiologist, CDCES’, Stanford Pediatric Endocrinology clinical team, members of Machine Learning Group at Stanford, and the Systems Utilization Research for Stanford Medicine (SURF) team. Although our current workflow is integrating specifically Garmin and Dexcom CGM data into TIDE, future work is underway and is aimed at increasing interoperability and integration of various types of CGM systems and commercially available activity trackers into the TIDE platform. This is being done by leveraging the Fast Healthcare Interoperability Resources^®^ (FHIR; a widely used standard for securely exchanging healthcare information electronically) software framework.

The existing TIDE dashboard shows a prioritized list of patients for weekly provider review with various CGM summary statistics (e.g., average glucose, percent CGM wear time, percent time-in-range, percent time below range, etc.), described elsewhere (15). When a CDCES selects a patient of interest, the glucose traces are displayed for the past two weeks. We are now extending this dashboard to incorporate additional physical activity metrics. The next iteration of TIDE will include the physical activity summary statistics (e.g., percent activity tracker wear time, average step count, etc.) as additional columns alongside the CGM summary statistics.

As a first step, our aim is to build out and use this physical activity and CGM overlay integration in TIDE for all 4T Exercise study participants before scaling the TIDE platform beyond Stanford’s borders. This would serve as incoming data to help healthcare providers and CDCES with tracking physical activity patterns and behaviors of newly diagnosed youth with T1D. Physical activity data in TIDE will include summary statistics to identify whether youth with T1D are meeting exercise guidelines (e.g., at least 60 minutes of moderate-to-vigorous physical activity per day or step count per day). Examples of these population-level flags will include percent activity tracker wear time, percent of exercise guidelines being met, and percent change in physical activity from the previous review period (e.g., every two weeks). Our goal with this novel data integration is that by increasing accessibility to physical activity data visualizations in TIDE, the CDCES and care team may be more willing to discuss exercise with their patients. Seeing graphic representations of activity data rather than relying on patient recall may help CDCES’ and the care team to make more informed decisions around insulin dose adjustments and overall diabetes care. Visualizations of physical activity data also offers another opportunity for healthcare providers to connect patients with specialists, where available (e.g., Certified Exercise Physiologist, etc.).

With the overlay of physical activity metrics and CGM data in TIDE, we are also working on specific “activity flags” that will highlight if rapid changes in glycemia occur during structured exercise bouts. For example, a ≥50 mg/dL drop or hypoglycemia (<70 mg/dL) during structured exercise will be flagged in the TIDE care model for review. This information may help healthcare providers understand whether dysglycemia occurs during exercise, but also determine the risk of nocturnal hypoglycemia with increased levels of physical activity (7, 26).

## Conclusions

At Stanford, we have developed a novel technology-enabled, telemedicine-based care model called Timely Interventions for Diabetes Excellence (TIDE) for patients with newly diagnosed T1D (14). This algorithm-enabled prioritization of T1D patients with CGM data for asynchronous remote review has improved CDCES’ workflow (i.e., reduced time spent per patient) and improved patients’ glucose time-in-range (14, 15).

Next steps include overlaying physical activity data (e.g., heart rate and step count) from wearable activity trackers with CGM data in the current TIDE care model. This activity-glucose multimodal algorithm-enabled decision-support tool will provide healthcare providers and CDCES’ with additional data to make more informed decisions around patient needs, insulin-dosing decisions, and overall diabetes management.

## Data availability statement

The original contributions presented in the study are included in the article. Further inquiries can be directed to the corresponding author.

## Ethics statement

The studies involving human participants were reviewed and approved by Stanford University's Research Compliance Office. Written informed consent/assent to participate in this study was provided by the participant and/or participant’s legal guardian.

## Author contributions

DZ devised the concept of the manuscript. DZ, RS, and DS wrote the initial manuscript and edits were provided by all co-authors. All authors contributed to the article and approved the submitted version.
